# Expanded carrier screening and preimplantation genetic diagnosis in a couple who delivered a baby affected with congenital factor VII deficiency

**DOI:** 10.1186/s12881-018-0525-9

**Published:** 2018-01-24

**Authors:** Wen-Bin He, Yue-Qiu Tan, Xiao Hu, Wen Li, Bo Xiong, Ke-Li Luo, Fei Gong, Guang-Xiu Lu, Ge Lin, Juan Du

**Affiliations:** 1 Institute of Reproductive and Stem Cell Engineering, Central South University, Changsha, Hunan 410078 People’s Republic of China; 2 Reproductive and Genetic Hospital of CITIC-Xiangya, Changsha, Hunan 410078 People’s Republic of China

**Keywords:** Expanded carrier screening, Preimplantation genetic diagnosis, Preimplantational genetic screening, Congenital FVII deficiency, Cystic fibrosis

## Abstract

**Background:**

Preimplantation genetic diagnosis (PGD) is a powerful tool for preventing the transmission of Mendelian disorders from generation to generation. However, PGD only can identify monogenically inherited diseases, but not other potential monogenic pathologies. We aimed to use PGD to deliver a healthy baby without congenital FVII deficiency or other common Mendelian diseases in a couple in which both individuals carried a deleterious mutation in the ***F7*** gene.

**Methods:**

After both members of the couple were confirmed to be carriers of the ***F7*** gene mutation by Sanger sequencing, expanded carrier screening (ECS) for 623 recessive inheritance diseases was performed to detect pathological mutations in other genes. PGD and preimplantational genetic screening (PGS) were employed to exclude monogenic disorders and aneuploidy for their embryos.

**Results:**

ECS using targeted capture sequencing technology revealed that the couple carried the heterozygous disease-causative mutations c.3659C > T (p.Thr1220Ile) and c.3209G > A (p.Arg1070Gln) in the ***CFTR*** gene. After PGD and PGS, one of their embryos that was free of congenital FVII deficiency, cystic fibrosis (CF) and aneuploidy was transferred, resulting in the birth of a healthy 3200 g male infant.

**Conclusion:**

We successfully implemented PGD for congenital FVII deficiency and PGD after ECS to exclude CF for the first time to the best of our knowledge. Our work significantly improved the reproductive outcome for the couple and provides a clear example of the use of ECS combined with PGD to avoid the delivery of offspring affected not only by identified monogenically inherited diseases but also by other potential monogenic pathologies and aneuploidy.

## Background

To date, more than 5000 Mendelian disorders have been identified in humans, including approximately 1300 autosomal and X-linked recessive disorders. Collectively, these Mendelian disorders account for 20% of infant mortality, approximately 79% of congenital anomalies and 10% of paediatric hospitalizations, thus imposing a significant burden on public health [1–3]. Preimplantation genetic diagnosis (PGD) is a powerful tool for preventing the transmission of Mendelian disorders from generation to generation. In the past two decades, there have been great advances in the technology and biopsy methods employed for PGD since its first successful application in 1990 [4, 5]. The number of single gene defects applied to PGD has been greater than 200, and the neonatal outcomes associated with PGD are comparable to those of intracytoplasmic sperm injection (ICSI) treatment [4, 6, 7].

However, the current PGD strategy for recessive disorders remains challenging to apply. Published data have shown that the main reasons that healthy couples seek PGD are a family history of a transmittable recessive disorder and the risk of passing disease-causative mutations to their offspring [8]. It is estimated that approximately 5 in 100 infertile couples are at risk of having a child affected with a recessive disorder, which might omit most high-risk carrier couples with an absence of a family history of a monogenic disorder [3]. Therefore, carrier screening for recessive diseases is an important component of preconception and prenatal care. Over the last 50 years, carrier screening has progressed from addressing a small group of highly selected diseases relevant to high-risk populations to an expanded list of diseases included in preconception and prenatal screening in all healthy individuals [9–11]. Theoretically, PGD can be expanded to any recessive genetic disease with a definitive molecular diagnosis.

Herein, we report a carrier couple who previously had adverse reproductive outcomes due to a deficiency of the *F7* gene. Expanded carrier screening (ECS) using targeted sequencing technology revealed that both members of the couple were carriers of pathogenic variants in the *CFTR* gene. We describe the successful application of ECS in combination with PGD and preimplantational genetic screening (PGS) to prevent the birth of offspring affected with FVII deficiency, cystic fibrosis (CF) or chromosomal abnormality. To the best of our knowledge, this is the first report of the application of PGD for FVII deficiency and the use of PGD and PGS for two monogenic disorders and aneuploidy screening simultaneously after preconception ECS.

## Methods

### Study subjects and clinical characterization

**Fig. 1:**
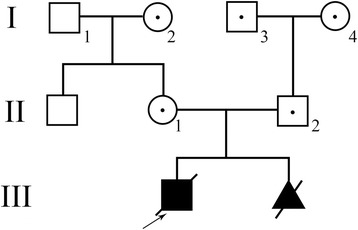
Pedigree structure of the family participating in this study. Affected members are indicated with filled symbols, and unaffected relatives are indicated by open symbols, whereas heterozygous carriers are indicated with a dot in the middle of the symbols. Numbers are allotted to the family members whose DNA samples were used in this study. The proband is indicated with an arrow

**Fig. 2:**
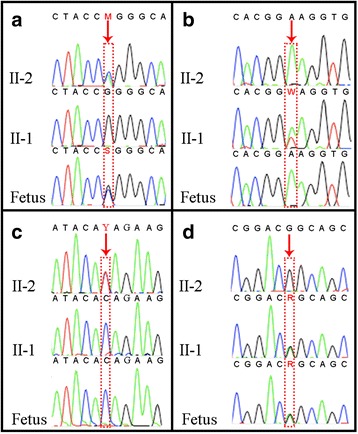
Sequencing of the couple’s DNA and amniocyte DNA. **a** The foetus harboured the c.1238G > C mutation in the *F7* gene inherited from his father (II-2), who is a carrier of the compound heterozygous mutations c.1238G > A and c.1238G > C in the *F7* gene. **b** The mother (II-1) harboured the heterozygous c.1126A > T *F7* mutation, but this mutation was absent in the foetus. **c** The father (II-2) harboured the heterozygous c.3659C > T mutation in the *CFTR* gene, but this mutation was absent in the foetus. **d** The foetus harboured the c.3209G > A mutation in the *CFTR* gene inherited from his mother (II-1). The red arrows indicate c.1238 and c.1126 in *F7,* and c.3659 and c.3209 in *CFTR*

A couple consisting of a 34-year-old woman and a 40-year-old man with normal phenotypes who had a proband child with congenital FVII deficiency and terminated a second pregnancy because of the same disease (Fig. 1) was referred to our centre (Reproductive and Genetic Hospital of CITIC-Xiangya). Haematological testing of the proband showed an obviously prolonged prothrombin time (24.1 s, reference range: 9.9–12.8 s), and testing showed 2.03% FVII activity (reference range: 70%–120%). Genetic testing via Sanger sequencing confirmed that the husband is a carrier of a c.1238G > C (p.Arg353Pro) mutation in the ***F7*** gene, while the wife is a carrier of a c.1126A > T (p.Lys316Term) mutation in the same gene. Furthermore, the husband was found to harbour another variation in the ***F7*** gene, c.1238G > A (p.Arg353Gln), which could mildly reduce factor VII activity (Fig. 2) [12, 13]. The couple sought PGD and PGS to prevent the recurrence of the FVII deficiency, other common Mendelian disorders and chromosomal abnormalities.

### Preconception expanded carrier screening of the couple

Both members of the couple gave their written informed consent, and genomic DNA was extracted from their blood samples for ECS using a commercial panel from BGI-Tianjin. The panel was designed to screen 547 genes associated with 623 monogenic disorders and includes recessive and X-linked diseases with severe and highly penetrant phenotypes as well as high-prevalence monogenic diseases with moderate phenotypes [3, 11]. The samples were sequenced (PE100) in one lane of the Hiseq2000 platform. Sequence data analysis was performed using a bioinformatics pipeline developed in a previous study [3]. Briefly, the Illumina analysis pipepline (CASAVA1.8) was employed for base calling. We then separated each barcoded dataset and removed the low-quality data using in-house scripts. The sequencing reads were subsequently aligned to the reference human genome (hg19) using the Burrows-Wheeler Aligner. Single-nucleotide variants and small insertions or deletions were identified using the Genome Analysis Toolkit (Broad Institute), while deletion or duplication of exons in genes was detected using an in-house pipeline. Variants were annotated with in-house scripts [3].

### IVF and biopsy

The couple underwent IVF treatment and biopsy as previously reported [14]. Briefly, after oocyte retrieval, mature oocytes were subjected to ICSI. On day 5 after fertilization, blastocysts in which trophectoderm cells had herniated out of the zona pellucida were chosen for biopsy.

### Mutation detection, linkage analysis, and comprehensive aneuploidy screening

Whole-genome amplification (WGA) of trophectoderm cells was performed using a commercial kit (REPLI-gMidi Kit, QIAGEN, Germany) according to the manufacturer’s instructions, with some modifications. ECS revealed that both members of the couple carried a heterozygous disease-causative mutation in the ***CFTR*** gene. PGD was performed by directly evaluating the ***F7*** and ***CFTR*** genes and via an embryo haplotyping strategy. Three pairs of primers were designed for direct mutation detection. For embryo genotyping, a set of short tandem repeat (STR) markers located on either side of the mutation was used to establish the paternal and maternal haplotypes. STR primers for one locus linked to the ***F7*** gene (D13S261) and three loci linked to the ***CFTR*** gene (D7S633, D7S480, and CFTR-IVS17) were amplified in separate singleplex polymerase chain reaction (PCR) assays using HotStarTaq DNA Polymerase (QIAGEN, Germany), and the results were detected via capillary electrophoresis on an ABI 3130 XL genetic analyser (Applied Biosystems, Foster City, CA, USA). The primer sequences are shown in Table 1. After PGD, embryos that were free of the monogenic disorders were also tested for chromosomal aneuploidy using the Ion PGM™ sequencing platform (Life Technologies, San Francisco, CA) according to a standard protocol. Briefly, the WGA products were purified with Agencourt® AMPure®XP beads (BeckMan), connected with an Ion Xpress™ Barcode (Life Tech), and subsequently sequenced on the Ion PGM™ platform (Life Tech), according to a standard protocol (https://ioncommunity.thermofisher.com/community/protocols-home). The raw sequencing data included approximately 0.5 M reads, which were mapped to the reference human genome (hg19) with a coverage rate of approximately 1%. Chromosomal copy number variation (CNV) analysis was performed for all samples using the Celloud cloud server (http://www.celloud.org/), offered by JBRH (Beijing, China). The applied analysis pipepline was similar to that in a previous study [15].

**Table 1:** Primer sets for mutation detection and linkage analysis

Primers	Primer sequence 5′-3’	
	Forward sequence	Reverse sequence
D13S261	CACCCTCAATCTCAACCCAC	GGAATGTGCTCTAATGCTGC
D7S633	TGAGCCTCGCATCACTGCAC	TCTGGGGAGTCCTTTAACAGTA
D7S480	TTCAGGTAGACAAGTTCCTGTC	TGGAGGGAGGAGAGTGGTAC
CFTR-IVS17	TGTCACCTCTTCATACTCATATTGG	AAACTTACCGACAAGAGGAACTCTG
F7-PGD	CTTCGTGCGCTTCTCATTGG	TTGCAGCCACTCGATGTACT
CFTR-17b	TTCAAAGAATGGCACCAGTGT	ATAACCTATAGAATGCAGCA
CFTR-19	ACAAATAGCAAGTGTTGCA	GCTTCAGGCTACTGGGATTC

### Confirmatory prenatal diagnosis and follow-up

Amniocentesis was performed at 19 weeks of gestation, and genomic DNA was extracted from cultured amniocytes using the QIAamp DNA Mini Kit (QIAGEN, Germany). Sanger sequencing was employed to confirm the absence or presence of the mutations in the ***F7*** and ***CFTR*** genes. Additionally, the chromosome karyotype was confirmed by amniocyte G-banding using the Automatic Chromosome Karyotyping System (ZEISS, Germany) following a standard protocol.

## Results

### Preconception expanded carrier screening

In total, 1.5 Gb of sequence was generated to achieve a mean coverage of 200×. After upstream filtering, only one disorder, cystic fibrosis, was found to show a high risk, as both members of the couple exhibited a mutation in ***CFTR***. A previously reported variation in the ***CFTR*** gene, c.3659C > T (p.Thr1220Ile), was identified in the husband. This mutation was also identified in a cystic fibrosis patient and was classified as a pathogenic mutation based on evaluation through in silico analyses [16]. The wife was also found to harbour a recurrent pathogenic mutation in the ***CFTR*** gene, c.3209G > A (p.Arg1070Gln) [17]. These variations were confirmed via Sanger sequencing (Fig. 2).

### Mutation detection and linkage analysis

Following ovarian stimulation, 24 oocytes were retrieved, 20 of which were fertilized through ICSI. Of these 20, 11 developed to the blastocyst stage with apparently normal phenotypes, and trophectoderm cells were collected for WGA. Sanger sequencing and STR analysis were performed on the whole-genome amplification products. The results of simultaneous PGD for congenital FVII deficiency and CF are summarized in Table 2. Embryos 1, 2, 6, 9, and 10 were affected with congenital FVII deficiency; embryos 4 and 5 were compound heterozygotes for the two *CFTR* gene mutations; embryos 7 and 8 were diagnosed as carriers of congenital FVII deficiency and CF; and embryo 3 was a carrier of congenital FVII deficiency but did not harbour either of the *CFTR* gene mutations. Due to severe allele drop out (ADO) for the ***F7*** gene and the linked STRs, the diagnosis of embryo 11 as either a carrier or patient was unclear.

**Table 2:** The results of mutation anlaysis and comprehensive aneuploidy screening

	*F7* gene				*CFTR* gene						PGS	Comment
Embryo ID	STR (bp, Maternal /Paternal)	Mutations		Interpretation	STR (bp, Maternal /Paternal)			Mutations		Interpretation		
	D13S261	c.1126 A > T	c.1238 G > C/A		D7S633	IVS17	D7S480	c.3209 G > A	c.3659 C > T			
1	173/171	T/A	G/A	Affected	171/167	134/129	156/150	G/G	T/C	Carrier	ND	
2	173/171	T/A	G/A	Affected	165/167	136/134	156/148	A/G	C/C	Carrier	ND	
3	171/171	A/A	G/A	Carrier	171/167	134/134	156/148	G/G	C/C	Normal	47,XY, + 16	Trisomy 16
4	171/171	AF	AF	AF	165/167	136/129	156/150	A/G	T/C	Affected	ND	
5	171/171	A/A	G/C	Carrier	165/167	136/129	156/150	A/G	T/C	Affected	ND	
6	173/171	T/A	G/A	Affected	161/167	134/134	156/148	G/G	C/C	Normal	ND	
7	171/171	A/A	G/C	Carrier	165/167	136/134	156/148	A/G	C/C	Carrier	46,XY	Transferred
8	171/171	A/A	G/C	Carrier	165/167	136/134	156/148	A/G	C/C	Carrier	47,XY, + 13	Trisomy 13
9	173/171	T/A	G/A	Affected	165/167	136/134	156/148	A/G	C/C	Carrier	ND	
10	173/171	T/A	G/C	Affected	165/167	136/134	156/148	A/G	C/C	Carrier	ND	
11	ADO /171	ADO /A	ADO/A	Uncertain	171/167	134/134	156/148	G/G	C/C	Normal	ND	

*AF* Amplification failed, *ND* No detection, *ADO* Allele drop-out

### Comprehensive aneuploidy screening

**Fig. 3:**
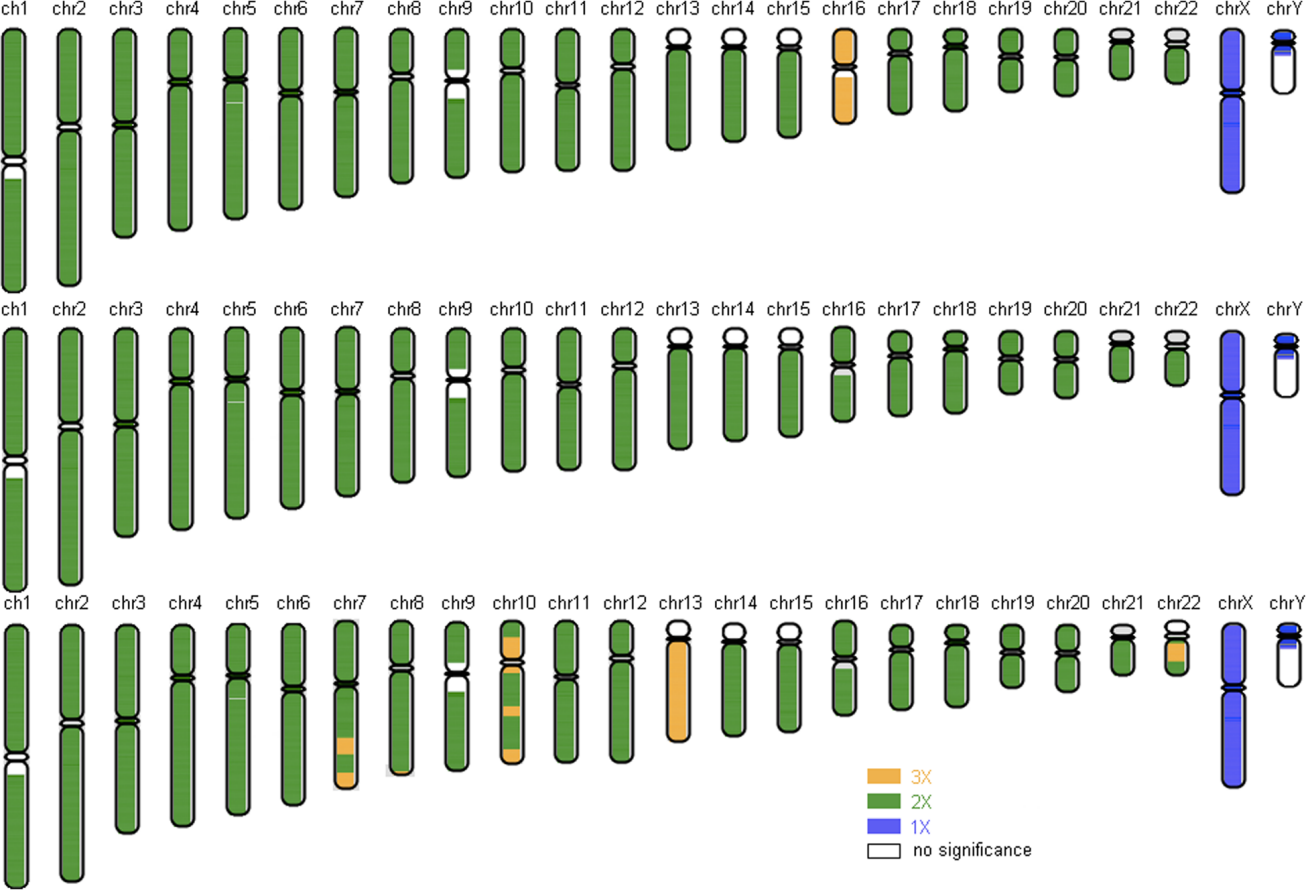
Comprehensive aneuploidy screening results for embryos 3 (upper), 7 (middle), and 8 (lower). White, blue, green, and yellow denote the copy numbers of the chromosome segments, which are shown on the lower right side. Embryo 7 was euploid, and embryos 3 and 8 exhibited trisomy 16 and trisomy 13*,* respectively

Embryos 3, 7, and 8 were chosen for screening chromosomal abnormalities since they were unaffected by congenital FVII deficiency and CF. Comprehensive aneuploidy screening revealed that embryo 7 (a carrier of congenital FVII deficiency and CF) was euploid, whereas trisomy 16 and trisomy 13 were identified in embryos 3 and 8, respectively (Fig. 3). Based on the results, embryo 7 was transferred.

### Confirmatory prenatal diagnosis and follow-up

Two weeks after embryo transfer, the woman showed a positive HCG pregnancy test, and over time, a single heartbeat and ongoing pregnancy were observed. The results of mutational and cytogenetic analyses of amniocyte DNA were in agreement with the original embryo 7 genotype (euploid and a carrier of congenital FVII deficiency and CF), and a healthy 3200 g male infant was born in October 2015 (Fig. 2). Further follow-up has shown that the boy is healthy.

## Discussion

In this study, a couple known to be carriers of FVII deficiency caused by mutations in the *F7* gene were referred to our hospital. ECS was employed to identify any additional inheritable diseases, and we subsequently performed aneuploidy testing in addition to PGD to ensure that a healthy embryo was transferred. A healthy infant was ultimately born, free of two monogenic disorders and chromosomal aneuploidy.

Congenital FVII deficiency and cystic fibrosis are life-threatening diseases. Congenital FVII deficiency caused by mutations in the ***F7*** gene is characterized by varying degrees of bleeding and is associated with easy bruising, recurrent epistaxis, menorrhagia, haemarthrosis, and intracranial haemorrhage [18, 19]. Cystic fibrosis is caused by mutations in the ***CFTR*** gene with manifestations of pancreatic insufficiency, pulmonary abnormalities such as recurrent and chronic bronchopulmonary infections, bronchiectasis, and chronic obstructive pulmonary disease, and congenital bilateral absence of the vas deferens (CBAVD), which causes male infertility [20, 21]. Our work helped the couple avoid the transmission of the cryptic recessive disease CF as well as the recurrent risk of congenital FVII deficiency.

The recessive carrier of cystic fibrosis was identified via ECS in this family; thus, the present study also highlights the importance of ECS in preventing genetic diseases in offspring. With the development of next-generation sequencing (NGS), an increasing number of studies have suggested the use of ECS to evaluate a large number of recessive disorders in healthy individuals, to reduce the risk of birth defects [22–24]. Preconception ECS allows couples to consider the complete range of reproductive options for preventing the birth of children affected by genetic diseases, including PGD, prenatal genetic testing, or the use of donor gametes. In this study, the couple was found to harbour causative mutations in the ***CFTR*** gene via ECS, which was unknown based on the results of previous examinations of the couple. The ECS results and reproductive outcome may contribute to genetic counselling and fertility guidance for other family members of the couple. Our findings illustrate that the combination of ECS with PGD presents broad potential future applications and can decrease the risk of not only known monogenically inherited diseases identified by family history but also other potential monogenic pathologies detected by ECS.

Furthermore, the present study confirmed the necessity of combining monogenic disorder detection (PGD) and aneuploidy screening (PGS) in women with advanced maternal age. A double-factor PGD protocol (PGD for monogenic diseases and PGS for chromosome abnormality) was previously proposed as a strategy for preventing a monogenic disease-free embryo with chromosomal aneuploidy from being transferred [25, 26]. In this study, the female patient was nearly 35 years old and exhibited a high risk of chromosomal aneuploidy. After the double-factor PGD, our data showed that 2 of the 3 embryos contained chromosomal aneuploidies. Thus, the importance of a PGD strategy accounting for both forms of genetic risk (monogenic disease and aneuploidy in embryos) was clearly demonstrated. Based on analysis and comparison of the results of PGD and PGS, we believe that performing PGS of unaffected embryos after monogenic disorder detection under a double-factor PGD protocol could decrease workload and cost. This strategy, of ECS followed by PGD and PGS, was employed in the couple evaluated in this study, resulting in a favourable reproductive outcome at a comparatively low cost. Most importantly, the two monogenic diseases were not transmitted to the offspring.

## Conclusions

In summary, we have successfully implemented PGD for congenital FVII deficiency for the first time using a combination of chromosomal, ***CFTR*** and ***F7*** gene analyses. If PGD were not performed, we would expect a 3/8 chance that the foetus would be unaffected by either condition (50% chance of being unaffected for F7 multiplied by a 75% chance of being unaffected by CF); thus, the use of PGD significantly improved the reproductive outcome for the couple in this case. This work-up provides a clear example of combining ECS with PGD to avoid offspring affected by not only identified monogenically inherited diseases but also other potential monogenic pathologies and aneuploidy. This strategy, of ECS followed by PGD and PGS, can successfully identify embryos suitable for transplantation, which may not only greatly reduce the risk of birth defects but also significantly improve pregnancy outcomes. Further studies are necessary to confirm our findings.

## Abbreviations

ADO: Allele drop out
CBAVD: Congenital bilateral absence of the vas deferens
CF: Cystic fibrosis
ECS: Expanded carrier screening
IVF: in vitro fertilization
NGS: Next-generation sequencing
PCR: Polymerase chain reactions
PGD: Preimplantation genetic diagnosis
PGS: Preimplantational genetic screening
STR: Short tandem repeat
WGA: Whole genome amplification
